# A small RNA regulates *pprM*, a modulator of pleiotropic proteins promoting DNA repair, in *Deinococcus radiodurans* under ionizing radiation

**DOI:** 10.1038/s41598-021-91335-8

**Published:** 2021-06-21

**Authors:** Jordan K. Villa, Runhua Han, Chen-Hsun Tsai, Angela Chen, Philip Sweet, Gabriela Franco, Respina Vaezian, Rok Tkavc, Michael J. Daly, Lydia M. Contreras

**Affiliations:** 1 Institute of Cellular and Molecular Biology, University of Texas at Austin, Austin, TX USA; 2 McKetta Department of Chemical Engineering, University of Texas at Austin, Austin, TX USA; 3 Department of Pathology, School of Medicine, Uniformed Services University of the Health Sciences, Bethesda, MD USA; 4 Henry M. Jackson Foundation for the Advancement of Military Medicine, Bethesda, MD USA; 5 Department of Molecular and Cellular Biology, School of Medicine, Uniformed Services University of the Health Sciences, Bethesda, MD USA; 6 Present Address: Department of Microbiology and Immunology, School of Medicine, Uniformed Services University of the Health Sciences, Bethesda, MD USA

**Keywords:** Small RNAs, DNA damage response, Bacteria

## Abstract

Networks of transcriptional and post-transcriptional regulators are critical for bacterial survival and adaptation to environmental stressors. While transcriptional regulators provide rapid activation and/or repression of a wide-network of genes, post-transcriptional regulators, such as small RNAs (sRNAs), are also important to fine-tune gene expression. However, the mechanisms of sRNAs remain poorly understood, especially in less-studied bacteria. *Deinococcus radiodurans* is a gram-positive bacterium resistant to extreme levels of ionizing radiation (IR). Although multiple unique regulatory systems (e.g., the Radiation and Desiccation Response (RDR)) have been identified in this organism, the role of post-transcriptional regulators has not been characterized within the IR response. In this study, we have characterized an sRNA, PprS (formerly Dsr2), as a post-transcriptional coordinator of IR recovery in *D. radiodurans*. PprS showed differential expression specifically under IR and knockdown of PprS resulted in reduced survival and growth under IR, suggesting its importance in regulating post-radiation recovery. We determined a number of potential RNA targets involved in several pathways including translation and DNA repair. Specifically, we confirmed that PprS binds within the coding region to stabilize the *pprM* (DR_0907) transcript, a RDR modulator. Overall, these results are the first to present an additional layer of sRNA-based control in DNA repair pathways associated with bacterial radioresistance.

## Introduction

Bacterial survival and adaptation to stressors requires a complex network of regulation woven by transcriptional repressors/activators, two-component systems, and post-transcriptional RNA regulators. Complex regulatory networks utilizing both transcriptional repressors/activators and post-transcriptional regulators such as small RNAs (sRNAs) have been discovered for a wide variety of stresses^1^. Examples of transcriptional regulators regulating or being regulated by sRNAs in *Escherichia coli* include: oxidative stress (OxyR, OxyS)^2^, acid stress (GadX, GadY)^3^, anaerobic stress (FNR/ArcA, FnrS)^4^, cell envelope stress (σ^E^, MicA/RybB/MicL)^5^, and metal ion homeostasis (Fur, RyhB)^6^. In each of these systems, integration of post-transcriptional regulation via sRNAs into these networks serves to fine-tune gene expression levels necessary for bacterial survival^7^.

sRNAs range in size from 50 to 500 nucleotides (nt) and typically do not encode functional proteins^1,8,9^. Instead, sRNAs regulate gene expression through antisense base-pairing interactions with target mRNA(s) that result in translational activation or repression by modifying the accessibility of the ribosome to the ribosome binding site (RBS) or alteration of mRNA stability^8–10^. Although some examples of sRNAs activating expression of their target mRNA are known, such as anti-antisense control or stabilization of mRNA by blocking ribonuclease (like RNase E) cleavage sites, a majority of sRNAs repress their mRNA targets^11^. Most sRNAs bind to the 5′ untranslated region (UTR) of the target mRNA, with some examples regulating at the 3′UTR and even fewer within the coding sequence^1,8,10–12^. Additionally, sRNAs are capable of regulating several mRNA targets, including transcriptional regulators^1,12^. In this manner, sRNAs are capable of regulating large networks of mRNAs in response to environmental and cellular signals and in coordination with transcriptional regulators^7^. As such, sRNAs provide a method of fine-tuning rapid responses to stresses given that they do not require translation for function and exhibit much shorter lifetime than the protein-based transcriptional regulators^1,12,13^.

Ionizing radiation (IR) represents an extreme environmental stress that damages molecular components directly and via the generation of reactive oxygen species (ROS), which results in DNA damage, genomic mutations, lipid peroxidation, and proteome oxidation^14^. While some transcriptional regulatory networks have been characterized^15^, post-transcriptional sRNA regulators involved in the IR stress response network have yet to be discovered. Additionally, the characterized regulatory networks have typically expanded on mechanisms of coordinating the upregulation of repair genes after stress, rather than the maintenance of these repair genes at lower expression levels during unstressed conditions^14,15^. However, discovery and characterization of sRNAs have that are involved in oxidative stress responses have remained limited to a few organisms^1^.

*Deinococcus radiodurans* is a gram-positive bacterium that is renowned for its unprecedented survival to gamma radiation (acute doses up to 20 kGy and chronic irradiation as high as 60 Gy/h), UV irradiation (up to 2000/Jm), desiccation, and other oxidative stresses^14,16,17^. During the extreme damage caused by these stresses, *D. radiodurans* protects its proteome, including the transcriptional and translational machinery, via small molecule Mn^2+^ antioxidants that scavenge cellular reactive oxygen species^18–20^. However, the regulatory network for radiation response remains to be fully characterized. Comparative genomics studies between *D. radiodurans* and radiation-sensitive organisms revealed very few differences in the type or number of DNA repair enzymes present^14,21^.

The coordinated repair of both damaged DNA and proteins after IR-induced damage requires a complex network of regulatory functions^21^. Three global transcriptional regulatory networks (regulated by DrRRA, DdrI, or PprI/DdrO) have been identified in *D. radiodurans*^17^*.* A two-component system involving the response regulator DrRRA (DR_2418) regulates catalase (*katA*/*katE1*/DR_1998) and other proteins involved in bacterial survival and metabolism^22,23^. The cAMP receptor protein (CRP), DdrI (DR_0977), regulates transcription of 18 genes (including lon protease (DR_0349)) in response to a variety of oxidative stresses and heat shock^24^. This DdrI-regulated network likely has an uncharacterized broader network as in silico predictions have proposed binding sites to 115 other candidate target genes^25^. A third unique transcriptional regulatory system to *Deinococcus* sp., termed the Radiation and Desiccation Response (RDR)^26^ controls expression of several DNA repair enzymes, including RecA (DR_2340, a key protein for DNA repair by homologous recombination)^27^ and PprA (DR_A0346, a pleiotropic protein involved in a multiprotein complex to stimulate DNA repair)^28,29^. Due to the importance of regulating DNA repair genes after IR-induced damage, the regulatory network contains several levels of regulation. On a basic level, the IR-activated protease PprI (DR_0167, also referred to as IrrE) de-represses the RDR regulon by cleaving DdrO (DR_2574), a promoter-binding repressor that recognizes the palindromic RDR motif (RDRM)^17,26,30–33^. At a different level of regulation, a cold-shock protein homolog, PprM (DR_0907) modulates PprA and KatA expression under unstressed conditions; PprM is also post-translationally modified via a PprI-dependent mechanism^34–36^.

Previous work from our lab identified 41 novel sRNAs in *D. radiodurans,* including 8 sRNAs that were differentially expressed during recovery post-exposure to acute IR^37^, suggesting that sRNAs provide additional fine-tuning of responses associated with recovery post-radiation exposure in *D. radiodurans.* In this study, we characterize one of these sRNAs, Dsr2, as a model for studying the role of sRNA-based regulation during recovery from IR as this sRNA exhibited the unique characteristic of being naturally differentially expressed during recovery from 10 kGy acute IR, but not under other oxidative stresses such as H_2_O_2_. Furthermore, knockdown of this sRNA results in a significant decrease in *D. radiodurans* survival and growth following acute (10 kGy) and chronic (57 Gy/h) IR exposures. Our results further demonstrate that Dsr2 is an integral piece of the radiation resistance network that had not yet been identified; specifically, it regulates the expression of stress response proteins (PprA and KatA) during unstressed conditions by stabilizing *pprM* via binding within the *pprM* coding region. Therefore, Dsr2 was renamed PprS (for sRNA regulator of pleiotropic proteins promoting DNA repair). Overall, we present the first characterized sRNA that plays a direct role during recovery from IR exposures.

## Results

### PprS is specifically differential expressed during recovery from IR

**Figure 1:**
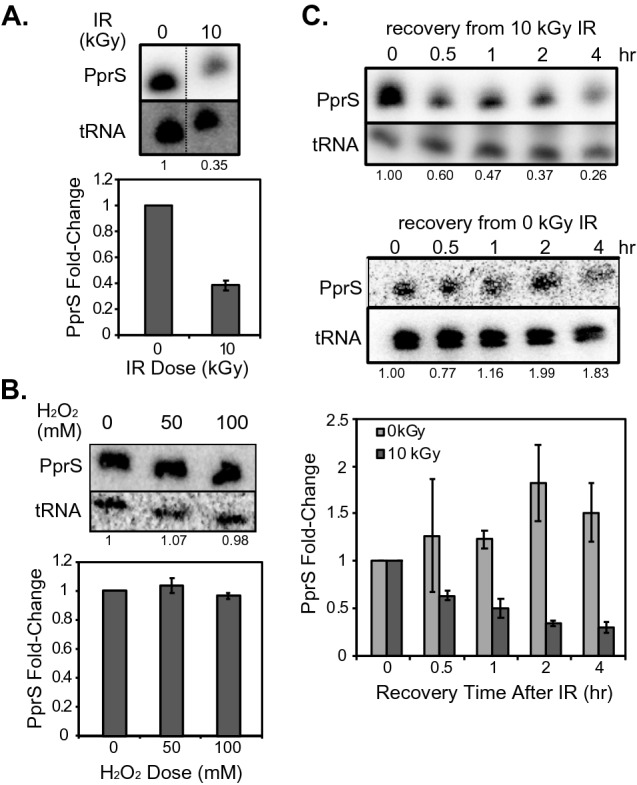
PprS demonstrates decreased expression under ionizing radiation, but not H_2_O_2_. (**A**–**C**) Representative Northern blotting images with bar graphs below demonstrating average fold-changes of PprS transcript levels (**B**) under 10 kGy of acute ionizing irradiation after 2 h recovery, (**C**) exposure to H_2_O_2_ stress (50 and 100 mM), and (**D**) after different recovery times from 10 kGy irradiation. Numbers below each blot represents the normalized fold change for that replicate (PprS band intensity normalized to tRNA loading control, then normalized to the unstressed data for the corresponding Northern blot). Northern blots have been cropped to show specific bands of interest, but are from the same membrane for each boxed figure. Error bars in bar plot demonstrate standard deviation from separate experiments (n = 2–3).

Among the 41 novel sRNA transcripts previously identified in *D. radiodurans* R1 by our lab*,* PprS (originally named Dsr2) is one of eight transcripts that is naturally differentially expressed during recovery from 15 kGy IR at both exponential and stationary growth phases^37^. PprS also exhibits decreased abundance during recovery from a less lethal IR dose (10 kGy) (Fig. 1A) equivalent to ~ 20% cell survival^38^; however, no changes in PprS transcript levels were observed after exposure to 50–100 mM H_2_O_2_ (Fig. 1B). This unique sensitivity to IR is unique to PprS as other sRNAs characterized in *D. radiodurans* (i.e. Dsr39 and Dsr18) were differentially expressed during recovery from IR as well as during H_2_O_2_ exposure^39^ or heat stress^40^, respectively.

Interestingly, differential expression of PprS occurred within 30 min of recovery from 10 kGy acute IR and persisted for at least 4 h after IR exposure (Fig. 1C). PprS transcript levels increased during the longer recovery times (2–4 h) as the cells reached stationary phase; we have observed previously^37^ that *PprS* is expressed at higher levels during stationary phase relative to exponential phase (Fig. 1C). To suggest possible regulatory pathways of PprS, we performed a shotgun proteomics time-course experiment to determine the dynamics of protein expression during recovery from 10 kGy acute IR. Similar to literature reports^41–49^, the response to IR in *D. radiodurans* is characterized by upregulation of DNA repair proteins (Fig. S1A), like DdrA (DR_0423), GyrB (DR_0906), and PprA (DR_A0346) of the RDR regulon after 1 h of post-IR recovery (Fig. S1B, Table S1). GO Enrichment analysis using PANTHER tools^50–52^ demonstrated significant enrichment of GO terms including metabolic process, DNA repair, and response to radiation (Table S2). During these recovery times, we observed that 25% of the 20 detected RDR-related proteins were significantly upregulated after 1 h of post-IR recovery, 45% after 2 h, and 50% by 4 h (Fig. S1B). In contrast, for the less characterized DrRRA and DdrI pathways, 15% of the 32 detected putative DdrI-related proteins^24,25^ and only 6% of the 78 detected putative DrRRA-related proteins^22,23^ demonstrated significant differential expression after 4 h post-IR recovery (Table S1). These results, combined with the timescale of PprS differential expression following IR exposure, suggested that PprS could be involved in radioresistance mechanisms and could be related to radiation response networks like the RDR pathway.

### Knockdown of PprS results in impaired growth and resistance to acute and chronic irradiation

To investigate the potential direct contribution of PprS to recovery from IR, we constructed a genetic knockdown of PprS (PprSKD) in *D. radiodurans* via homologous recombination and confirmed the strain via genomic PCR (Fig. S2A). Although a complete PprS deletion was attempted, a homogenous deletion of PprS could not be achieved, suggesting that PprS could be essential for cell viability. However, the knockdown strain demonstrated at least a twofold decrease in PprS expression as compared to the wild-type R1 strain (WT), as revealed by Northern blotting analysis (Fig. S2B).

**Figure 2:**
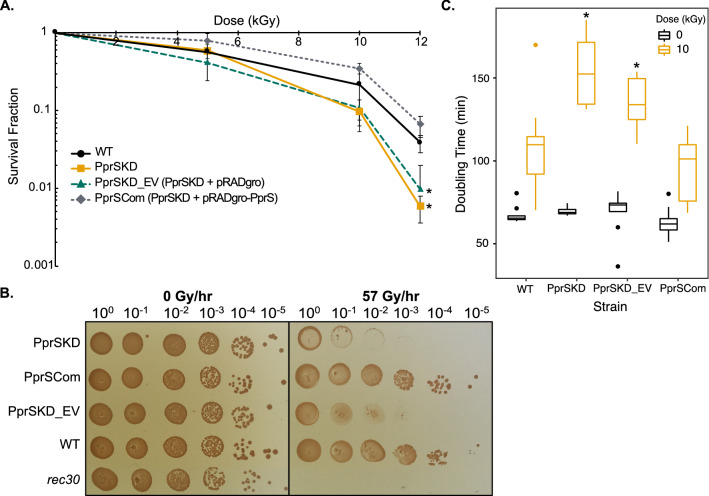
PprSKD showed reduced survival to acute IR and reduced growth under both acute and chronic IR. (**A**) Survival curve of *D. radiodurans* PprS knockdown mutant (PprSKD), PprSKD with empty plasmid control (PprSKD_EV), PprS complementation (PprSCom) and WT at different doses of acute IR. Error bars represent standard deviation of biological triplicates and *p-value < 0.05 between the strain compared to WT by two-tailed Student’s t-test. (B) Growth of WT, PprS knockdown and complement strains, and a *recA* defect strain (rec30) under chronic irradiation at 57 Gy/h for 5 days. Numbers over the images indicate the dilution ratio of colonies. (**C**) Doubling time of biological triplicates of WT and PprS knockdown and complement strains during recovery from 10 kGy acute IR or sham exposures. Knockdown of PprS does not impact *D. radiodurans* growth phenotype during unirradiated conditions. However, PprSKD demonstrates a significant reduction in growth rate during recovery from IR compared to WT. This growth defect after IR is lost after PprS complementation. *p-value < 0.05 for each comparison by two-tailed Student’s t-test.

*Deinococcus radiodurans* PprSKD exhibited a significant (*p*-value < 0.05) decrease in survival compared to the WT strain post-exposure to 12 kGy acute IR (Fig. 2A). A decrease in growth of the PprSKD strain was also observed under chronic IR (57 Gy/h for 5 days) (Fig. 2B). In addition to the reduction in survival, PprSKD demonstrated a significant reduction in doubling time during recovery from IR compared to WT, but not under sham (no radiation) conditions (Fig. 2C and Fig. S3A). To determine if this phenotype was a direct effect of the reduced level of the PprS sRNA, we constructed a complementation strain by constitutively expressing PprS from the pRADgro plasmid^58^ (pRADgro-PprS) within the PprSKD strain (referred to as PprSCom). While the empty vector control (PprSKD + pRADgro (PprSKD_EV)) displayed a survival phenotype comparable to the PprSKD strain, the complementation strain recovered the radioresistance phenotype similar to WT for acute IR (Fig. 2A) and growth under chronic IR (Fig. 2B) or after acute IR (Fig. 2C and Fig. S3A). In addition to the changes in doubling time between the strains recovering from IR exposure (Fig. 2C), we also observed a difference in the lag time; PprSKD and PprSKD_EV demonstrated a significant increase in lag time following IR exposure as compared to the WT or PprSCom *D. radiodurans* strains (Fig. S3B). After IR exposure, growth arrest permits DNA repair to occur in *D. radiodurans*^14,53^. Therefore, this increase in lag time in the PprSKD strain suggests a deficiency in the dynamics of DNA repair which could contribute to the radiation sensitivity observed (Fig. 2 and Fig. S3).

To validate that the effect on IR survival was unique to PprS, we created knockout (KO) or knockdown (KD) strains of seven sRNAs (Dsr5KO, Dsr6KD, Dsr9KD, Dsr17KD, Dsr18KO, Dsr19KD and Dsr39KO) and confirmed strain construction by genomic PCR (Fig. S4A); we previously demonstrated these sRNA were differential expressed under IR or during recovery from other oxidative stresses or found that these were highly conserved among *Deinococcus* species^37^. Importantly, under acute IR, none of these KO/KD strains exhibited any significant differences in survival compared to WT (Fig. S4B). Whereas other sRNAs (Dsr11, Dsr18, Dsr39, Qpr6, and OsiA) in *D. radiodurans* have been characterized as important regulators under other stresses (i.e. H_2_O_2_ and heat stress)^39,40,54–56^, PprS has been the only sRNA identified to impact survival and growth uniquely post-IR exposure. These results suggest that PprS is unique among known sRNAs in *D. radiodurans* in its contribution to IR recovery.

### PprS activates the expression of *pprM* (DR_0907) by binding within the coding sequence to stabilize the transcript

To identify potential regulatory targets of PprS important for recovery from IR in *D. radiodurans*, we conducted MS2-affinity purification coupled with RNA sequencing (MAPS)^57^. Two MS2 binding domains (MS2BD) were fused to the 5′ end of PprS and constitutively expressed off the pRADgro plasmid^58^ in wild-type *D. radiodurans* R1*.* RNA-sequencing of the MS2-co-immunoprecipitated RNA samples identified 135 transcripts at least twofold significantly (adjusted *p*-value < 0.05) enriched, compared to the MS2-only construct (Table S3). These putative targets are involved in a variety of pathways including translation, transcription, ribosome biogenesis and DNA repair/replication (Fig. S5A). GO terms for translation (5.08-fold) and gene expression (2.88-fold) were significantly enriched (Table S2) by GO Enrichment analysis^50–52^. Supporting the role of PprS within the radiation response, we observed significant enrichment of three DrRRA regulated transcripts (DR_0944 (thioredoxin) and two uncharacterized proteins (DR_1264 and DR_1263)) and *pprM* (DR_0907) among the top 8 targets (at least fourfold enriched) (Fig. S5B). PprM is a predicted cold shock protein that has been described previously as being a modulator of the RDR response network^34^. It is worth noting that deletion of PprM has been shown to decrease survival of *D. radiodurans* post-exposure to IR, suggesting an important role of PprM in mechanisms for radiation recovery^34^. After predicting putative binding sites using the prediction software, IntaRNA^59^ (Fig. S6), we focused our analysis on *pprM*, which, in addition to its relation to radioresistance, had the lowest predicted binding energy within the top 5 MAPS enriched candidates of PprS.

**Figure 3:**
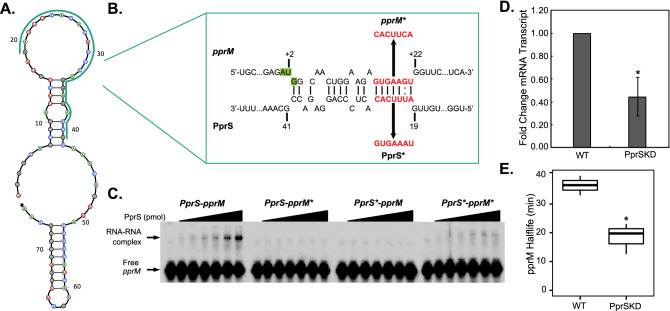
PprS binding stabilizes *pprM* transcript. (**A**) Predicted structure of PprS (from NUPACK^61^) with region of PprS-*pprM* interaction highlighted. (**B**) Prediction of PprS binding to *pprM* by IntaRNA with mutation of *pprM*-PprS interaction site shown in red. Nucleotide positions are given for the revised start codon of *pprM*. (If utilize the start codon annotated in the *D. radiodurans* genome, the predicted binding site is from + 149 to + 163 nt after the start codon). (**C**) Representative EMSA (n = 2–3) of PprS binding to *pprM* transcript in vitro. In each lane, 1 pmol of radiolabeled *pprM* mRNA fragment (− 33 to + 100 nt relative to start codon) was incubated with 0–100 pmol of PprS sRNA. K_d_’s were determined for the PprS-*pprM* EMSA to be 39.59 ± 15.56 pmol and 45.59 ± 1.84 pmol for the complemented mutations (PprS*-pprM*). (**D**) *pprM* transcripts levels were determined in unstressed conditions by RT-qPCR analysis. Bars represent the fold changes in gene expression compared to WT. Error bars represent standard deviation of triplicate biological samples. Difference in *pprM* transcript level between strains is significant by two-tailed Student’s t-test (*p*-value < 0.05). (**E**) Boxplot of *pprM* halflife in WT versus PprSKD strains calculated from Northern blot analysis. Difference between half-lives (36.46 ± 3.37 min for R1 versus 18.48 ± 5.37 min) was significant by two-tailed Student’s t-test (*p*-value < 0.05) from biological triplicates from two independent experiments (representative Northern blot presented in Figs. S8 and S10).

We next sought to further characterize the interaction between PprS and *pprM*. IntaRNA^59^ identified a putative binding site on one of PprS’s predicted stem loops (Fig. 3A) that interacts with a region ~ 149 nt inside the coding region of *pprM* (Fig. 3B). However, *pprM* has been reported to be incorrectly annotated in the *D. radiodurans* genome. Literature proteomics data^60^ locates the actual start codon 141 nt downstream the annotated start codon. Our 5′RACE analysis suggests the TSS at 120 nt downstream (producing a 21 nt long 5′UTR). Utilizing the corrected annotation position, PprS is predicted to bind from + 3 to + 21 nt after the start codon (Fig. 3B). This prediction was surprising considering most sRNAs bind towards the 5′ end of the target mRNA^1,10^. To validate the potential binding of PprS to the *pprM* transcript, we performed an electrophoretic mobility shift assay (EMSA). Indeed, binding of PprS to the *pprM* transcript was verified in vitro by EMSA (K_d_ = 39.59 ± 15.56 pmol) (Fig. 3C). Mutations to the predicted binding region on either PprS or *pprM* eliminated formation of the RNA-RNA complex (Fig. 3B,C); however, RNA-RNA complex formation was recapitulated in a compensatory interaction between the two mutated RNAs (K_d_ = 45.59 ± 1.84 pmol) (Fig. 3C), verifying the direct interaction between PprS and *pprM* mediated through the predicted binding site (Fig. 3A,B).

Comparison of the protein expression between *D. radiodurans* WT and PprSKD revealed a decrease in PprM protein levels in the PprSKD strain compared to WT (Fig. S7A, Table S4), suggesting an activation mechanism of regulation of PprS on *pprM*. To differentiate between the different mechanisms of activation (i.e., altering accessibility of the ribosome binding site versus increasing mRNA stability), we examined the *pprM* transcript levels by RT-qPCR (Fig. 3D). The transcript levels of *pprM* were > twofold decreased in the PprSKD strain as compared to WT *D. radiodurans* (Fig. 3D). Indeed, the *pprM* transcript half-life in PprSKD (18.48 ± 5.37 min) was significantly shorter than in WT (36.46 ± 3.37 min), supporting this model in which PprS stabilizes the *pprM* transcript in vivo (Fig. 3E and Fig. S8). As a final confirmation of this model, we sought to determine the in vivo regulatory effect on native levels of *pprM* after inducing expression of PprS*.* We expressed PprS from an inducible p_Spac_ promoter^62^ in *D. radiodurans* PprSKD and detected native *pprM* transcript levels by Northern blotting analysis. Slight induction of PprS resulted in an increase in *pprM* transcript levels (Fig. S7B), reinforcing the positive regulatory effect of PprS on *pprM.* From these experiments we propose that the PprS sRNA binds to the coding region of *pprM* to promote its stabilization during unirradiated conditions.

### PprS modulates stress response networks through *pprM* in *D. radiodurans *(and likely affects a broader set of targets beyond *pprM*)

**Figure 4:**
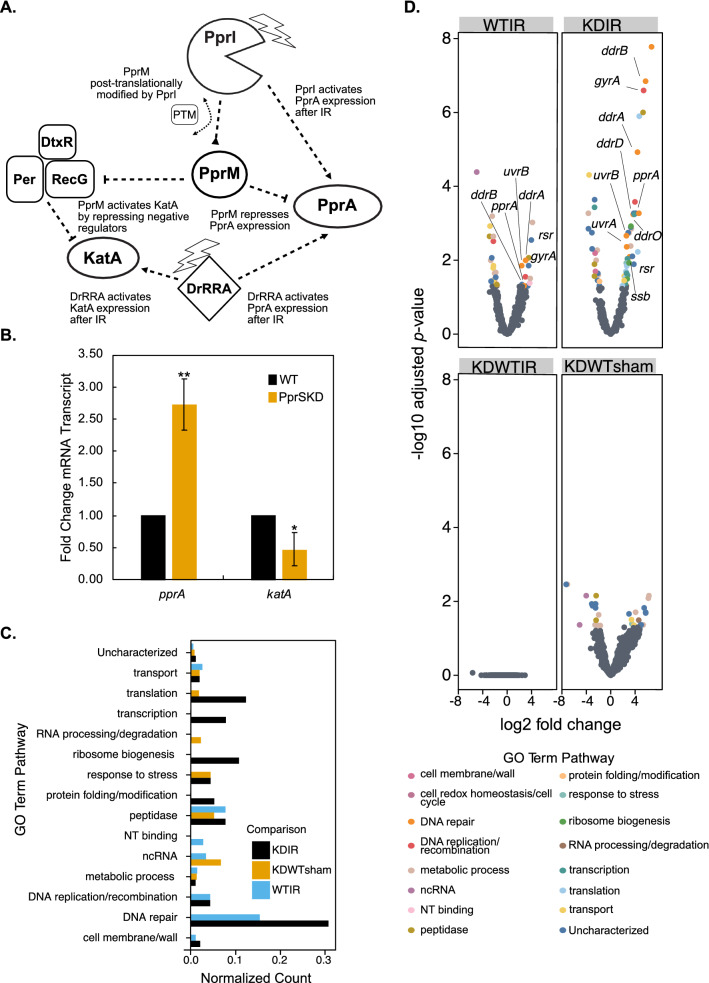
PprS stabilization of *pprM* affects *pprA* and *katA* levels under unstressed conditions and alters metabolic pathways whilst retaining RDR upregulation during recovery from IR. (**A**) Regulatory network coordinated by PprM as based on the literature. (**B**) *pprA* and *katA* transcripts levels were determined in unstressed conditions by RT-qPCR analysis. Bars represent the fold changes in gene expression compared to WT. Error bars represent standard deviation of triplicate biological samples. Difference in expression between strains is significant by two-tailed Student’s t-test (*p-value < 0.05, **p-value < 0.005). (**C**, **D**) Transcriptomics analysis demonstrated similar response of RDR pathway during recovery from 10 kGy of IR in PprSKD compared WT, in contrast to other metabolic pathways. (**C**) Normalized count of GO Term pathways represented in the significantly differentially expressed transcripts for each comparison. Normalized count was calculated by normalizing the number of significantly differentially expressed proteins in each GO Term pathway to the total number of proteins within that term. (**D**) Volcano plot of significant differentially (adjusted *p*-value (*p*-adj) < 0.05 and log2 fold-change > 1 or < − 1) expressed transcripts colored by GO Term pathway (RDR pathway labeled with text) from biological triplicate samples. Specifically, 34 transcripts were significantly differentially expressed in the WT strain during recovery from 10 kGy IR compared to sham conditions (WTIR). In the PprSKD strain, 61 transcripts were significantly differentially expressed compared to sham conditions (KDIR). However, no transcripts were significantly differentially expressed between the two strains during post-IR recovery (KDWTIR). Comparing the two strains under sham (no radiation) conditions (KDWTsham) demonstrated 31 significantly differentially expressed transcripts.

The model that PprM regulates other proteins involved in *D. radiodurans* stress response, such as PprA and KatA^34–36,60^ (Fig. 4A), suggests that PprS could subsequently affect a larger regulatory network. To test if PprS indirectly affects this broader pathway through PprM, we performed targeted RT-qPCR of *pprA* and *katA* transcriptional expression in PprSKD under sham conditions. The decreased *pprM* expression in the PprSKD strain (due to the reduced stabilization of *pprM* by PprS (Fig. 3D,E and Fig. S7)) also results in alteration to the transcripts that PprM modulates (i.e., *pprA* and *katA*). We observed a significant increase in *pprA* expression and a decrease in *katA* levels in the PprsKD strain (Fig. 4B), as had been previously observed after deletion of *pprM* (and suggested by model in Fig. 4A)^34,35^. This data supports a model in which PprS represses PprA and activates KatA during unstressed conditions by directly stabilizing the modulator *pprM*.

Given the direct effect of PprS on *pprM* stability, and thereby its indirect effect on the broader PprM network (i.e., *pprA* and *katA*) (Fig. 4A), we next analyzed the transcriptome-wide impact of the PprS knockdown. In previous studies, deletion of PprM results in global changes of gene expression patterns within multiple pathways (i.e. metabolism and protein homeostasis)^36^; these alterations to these house-keeping pathways has ultimately been hypothesized to underlie the observed survival decrease to IR in *D. radiodurans* in the absence of PprM^34^. Consistent with the hypothesis that PprS has a broader effect due to its stabilization of *pprM,* we observe a similar pattern in the PprSKD transcriptome. For instance, both when PprS and PprM are knocked down/out we see significant changes in translation, metabolic processes, and response to stress pathways during unstressed conditions (Fig. 4C,D, Table S5). Despite the effect of PprM on these house-keeping pathways, deletion of PprM does not change the upregulation of the RDR regulon during recovery from IR^34,36^. Similarly, knockdown of PprS does not result in any change to the upregulation of the RDR genes such as *recA* (DR_2340), *pprA* (DR_A0346) and *gyrA* (DR_1913)^42,44,45^ following 10 kGy acute IR exposure (Fig. 4D, Tables S6, S7). Similar to the unstressed conditions, we observed significant enrichment of other pathways including translation, transcription, and RNA biosynthetic process for the PprSKD strain recovering from IR (Fig. 4C, Table S2). Overall, these results support the model of PprS affecting a larger range of targets in *D. radiodurans* by stabilizing *pprM* during unstressed conditions.

## Discussion

**Figure 5:**
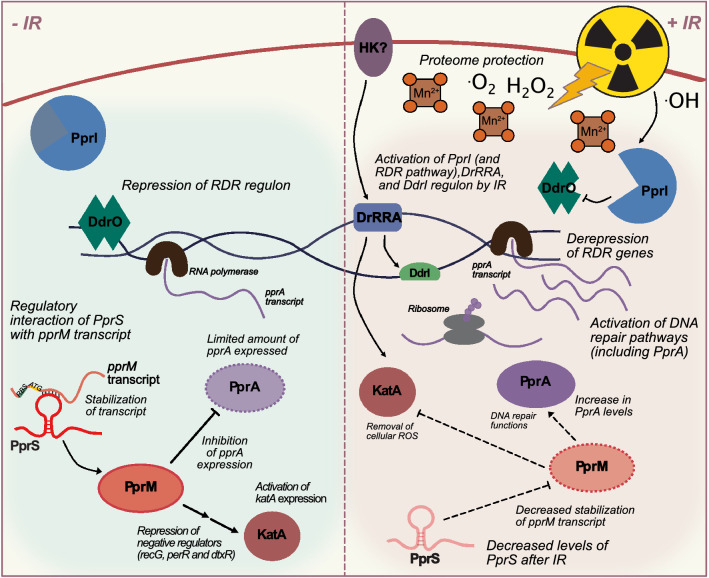
Model of PprS Regulatory Network in *D. radiodurans.* Three main transcriptional regulators have been identified in *D. radiodurans*: (1) a two-component system composed of the response regulator (DrRRA) and an unidentified histidine kinase (HK), (2) CRP regulator DdrI activated by DrRRA, and (3) the Radiation and Desiccation Response (RDR) containing the repressor protein DdrO and the IR-activated protease PprI. PprS is expressed during unirradiated conditions and binds to the *pprM* transcript within its CDS, stabilizing the transcript. PprM represses PprA expression to maintain low levels of PprA during unstressed conditions, and represses negative regulators of KatA. During recovery from IR, when PprA is required for DNA repair processes, transcriptional and translational machinery is protected by Mn-antioxidants and transcriptional regulators (PprI, DrRRA, and DdrI) upregulate critical cellular repair genes, including PprA and KatA. PprS transcript levels are reduced, which results in the destabilization of *pprM* and the corresponding activation of PprA expression.

In this study, we present the first examination of sRNA-driven regulation during recovery from IR, and highlight the interconnected regulatory networks driving bacterial survival. We demonstrate that a newly characterized sRNA, PprS, is vital to radiation resistance mechanisms in *D. radiodurans* based on the detrimental effect on survival and growth under IR that we observe even when PprS is knocked down (PprSKD) (Fig. 2). Furthermore, we identified and characterized one of PprS’s mRNA targets, *pprM,* that is stabilized by an uncommon sRNA regulatory mechanism in which PprS binds within the coding region to stabilize *pprM* (Fig. 3). To our knowledge, PprS is the first sRNA implicated in bacterial radioresistance. Importantly, we propose its function as a part of the network of global regulators involved in radiation resistance (Fig. 5). Through this model, we propose that the sensitivity to ionizing radiation observed after knockdown of PprS is largely due to its regulatory effect on the modulator protein, PprM. By disrupting the native regulation of DNA repair enzyme (PprA) and catalase (KatA) exerted by the PprM protein, the knockdown of PprS hinders the ability of *D. radiodurans* to immediately respond and repair ionizing radiation induced damages. Additionally, disruption of other house-keeping pathways (as also observed upon PprM deletion^34^) could also make *D. radiodurans* more vulnerable to IR stress. Our results support the growing field that post-transcriptional regulation by sRNAs offers an additional layer in coordinating and fine-tuning complex environmental stress responses in bacteria.

As part of our model, we propose PprS binds within the coding sequence of its mRNA target to activate gene expression. Although several sRNAs activate expression of their target, a majority function via repression^11^. For those sRNAs with activation mechanisms, most bind to the 5′UTR of the target mRNA to block degradation by RNases, or release occlusion of the RBS by anti-antisense control^11^. As suggested by currently characterized sRNA mechanisms, the binding of PprS to *pprM* + 16 nt after the start codon would typically repress the mRNA target expression by blocking translation^1,9–11^. However, our data strongly demonstrates an activation mechanism via stabilization of *pprM*. Considering the roles of RNases in RNA degradation is largely unknown in *D. radiodurans*, further detailed studies of the exact mechanism of PprS regulation on *pprM* are needed in the future.

Importantly, PprS is not the only sRNA to function through this type of activation mechanism; it has been reported that the SgrS sRNA in *E. coli* binds within the coding sequence of the co-transcribed upstream gene, *pldB*, to block RNase E truncation of the *pldB-yigL* mRNA^63^. We hypothesize that the binding of PprS to *pprM* results in protection of the mRNA transcript from degradation. Interestingly, the 5′UTR of *pprM* is relatively short (only 21 nt long) which could present physical limitations for PprS to bind the 5′UTR without occluding the RBS. Binding of PprS within the coding sequence of *pprM* could provide protection from RNase degradation of the transcript, whilst still leaving the RBS available. Short 5′UTRs or leaderless mRNAs have been demonstrated to be common in the transcriptome of *D. deserti*^64^, suggesting the possibility of more sRNAs functioning through this mechanism in *Deinococcus* species. Although more experiments are necessary to determine the precise molecular details, we suggest that PprS represents another example of a sRNA that activates its mRNA target via binding within the coding sequence.

It should also be noted that tools and promoters in *D. radiodurans* are still extremely limited which challenged our in vivo characterization approaches to validate this activation mechanism of PprS on *pprM*. Currently, there is one inducible promoter (p_Spac_) available in *D. radiodurans*; however, induction times of approximately 16 h are required for measurable increases in expression (as previously reported^62^). We attempted to construct translational fusion reporters, however, global effects of PprS knockdown (smaller size and lower fluorescence of all fluorescent constructs tested (Fig. S9)) combined with the weak induction of PprS from the p_Spac_ promoter challenged these assays. However, we were able to demonstrate this activation mechanism of PprS on the *pprM* transcript through a variety of other experiments.

Similar to other bacterial stress responses, the RDR network represents a regulatory system critical to the coordination of radioresistance genes. Importantly, our results are the first to present an additional layer of sRNA-based control within RDR-regulated genes^26^. The regulation of *pprM* by PprS results in modulation of expression of other genes, like PprA, in the RDR network, which are in turn regulated by other transcriptional regulators and protein-level modulators. The identification of a role of sRNAs as modulating modulators of the RDR network of *D. radiodurans* further increases the complexity of this phenotype. Add to this, there is mounting evidence across the tree of life that IR resistance is a polyphyletic metabolic trait^65,66^. Among yeasts, bacteria, archaea and simple animals (rotifers), the most IR-resistant representatives consistently hyperaccumulate Mn antioxidants. This suggests that studying the response of sRNAs in cells under different nutrient conditions could help to further define their mode of action within highly complex regulatory webs.

Based on our data, we propose a model (Fig. 5) summarizing the network of radiation response in *D. radiodurans* in which, during sham conditions, *pprM* is stabilized by PprS, resulting in fine-tuning of PprA and KatA by PprM. PprM modulates KatA expression by suppressing *katA* negative regulators (*recG, per*, and *dtxR*), but does not affect expression of the positive regulator of *katA*, DrRRA (Fig. 4A)^35^. In a similar network, PprM suppresses expression of PprA under unstressed conditions^34^ in a PprI-dependent manner (Fig. 4A). PprA has been reported to be regulated by PprI, PprM, and DrRRA^22,34,67^. PprA is involved in DNA repair with GyrA (DR_1913) and is thought to aid chromosomal positioning in DNA segregation during DNA repair and cell division as demonstrated by the significant decrease in survival to IR and faulty chromosomal separation during recovery from IR in a *pprA* knockout strain^28,68^. This regulatory effect on PprA by PprM has been suggested to be due to the RNA-binding ability of PprM^60^ which could be influenced by the post-translational modification of PprM by PprI^34^. It is worth noting that the regulatory mechanism of PprM remains to be fully elucidated including its precise biochemical function and full regulatory network^34,36,60,69^. Thus far, the importance of PprM to radiation recovery can be attributed to its regulation of PprA and KatA^34,35^, however a wider network is also likely. As many sRNAs target more than one mRNA^8^, we hypothesize that further characterization of PprS and its mRNA targets (including the other top 7 targets as predicted by MAPS (Table S3)) will provide additional insight into its regulatory mechanism of radiation recovery in *D. radiodurans.*

In summary, we propose PprS as a global regulator that interacts with radiation response pathways including the RDR regulon to mediate cross-talk between the different radiation response networks via its stabilization of *pprM* in unirradiated conditions (Fig. 5). Due to the importance of tightly regulating DNA repair and catalases, disruption of this network (such as by decreased PprS expression) results in an increase in radiosensitivity of *D. radiodurans.* Our study demonstrates an example of the interconnected regulation coordinated by transcriptional regulators, protein modulators, and post-transcriptional regulators in response to environmental stressors in bacteria*.*

## Materials and methods

### Bacterial growth and stress conditions

*Deinococcus radiodurans* strain R1 (ATCC 13,939) was grown at 32 °C in TGY broth (1% tryptone/0.1% glucose/0.5% yeast extract) or TGY solid medium. All strains are listed in Table S8 and primers used for Northern blotting, cloning and sRNA deletions are listed in Table S9. For constructing deletion mutants, antibiotics were used at the following concentrations: 100 μg/ml ampicillin (*E. coli*); 25 μg/ml (*E. coli*) or 3.4 μg/ml (*D. radiodurans*) chloramphenicol; and 32 μg/ml (*E. coli*) or 16 μg/ml (*D. radiodurans*) kanamycin. For hydrogen peroxide exposure, cells were grown to exponential phase (optical density at 600 nm [OD_600_] = 0.8) and incubated with 0–100 mM of hydrogen peroxide (Fisher Scientific) at 4 °C for an hour. No additional recovery time was provided for the cells after H_2_O_2_ exposure as performed previously for detection of sRNA differential expression^39^. The cell pellets were then collected and used for RNA extraction. For acute IR exposures, exponential phase cells (OD_600_ = 0.8) were irradiated with a 10-MeV, 18-kW linear accelerator (LINAC) β-ray source at dosages ranging from 0 to 15 kGy (250 Gy/s) at the National Center for Electron Beam Research, Texas A&M University as reported previously^37,38^. Irradiated samples were diluted twofold in fresh TGY media for 0 to 6 h of recovery (for time-course Northern blotting and proteomics experiment) or 2 h (for Northern blotting assay and transcriptomics) at 32 °C immediately following irradiation and used for RNA preparation.

### Construction of sRNA deletion/knockdown strains in *D. radiodurans*

To delete sRNA coding regions from the genomes of *D. radiodurans*, we used a suicide plasmid to introduce the desired interruption by homologous recombination as previously reported^56^. Briefly, the upstream and downstream homologous regions of the sRNA to be deleted were cloned into pUC19mPheS plasmid which contains kanamycin resistance, lox66 and lox71 sequences. Double cross-over homologous recombination occurred after transformation of the plasmid into *D. radiodurans* R1. Mutant strains were selected on TGY agar plates with kanamycin and 4-chloro-phenylalanine (5 mM, Sigma-Aldrich) for five rounds of selection. This process selects for cells that had undergone homologous recombination to replace the target region with the kanamycin cassette and no longer express the pUC19mPheS plasmid. The antibiotic resistance marker was removed using Cre-Lox recombination (expressed off the pDeinoCre plasmid). Deletion of the desired sRNA was confirmed with both genomic PCR and Northern blotting analysis. The primers used for deletions are listed in Table S9.

### Survival and growth assays during recovery post-IR

For survival to acute IR exposures, biological triplicates of exponential phase cells (OD_600_ = 0.8) were irradiated with a 10-MeV, 18-kW linear accelerator (LINAC) β-ray source at doses 0, 5, 10, 12 kGy (250 Gy/s) at the National Center for Electron Beam Research, Texas A&M University as reported previously^37,38^. Irradiated samples were kept static on dry ice during transport to and from the irradiator (~ 2 h each way). Following irradiation at room-temperature, samples were immediately serially diluted (10^–0^ to 10^–6^) and plated onto TGY agar plates. Samples were grown at 32˚C for 3 days before colony counting. For the growth curve, samples were diluted to OD_600_ = 0.3 into fresh TGY liquid media and run for 24 h at 32˚C, 200 rpm in a BioScreen C (Growth Curves USA). Doubling times and lag times were determined using R package GrowthRates^70^. The “fit_easylinear” function calculates maximum growth rates from the log-linear part of a growth curve; this function also provides the lag time by estimating the intersection between the fit and the horizontal line equal to the first absorbance value^70^.

Resistance to chronic gamma radiation was assessed by first incubating single colonies of *D. radiodurans* strains R1 (wild-type), Rec30 (*recA* defect mutant), PprSKD, PprSKD_EV, and PprSCom in 10 mL of liquid TGY medium overnight at 32 °C, 180 rpm. The next day, cultures were adjusted to OD_600_ = 0.8, serially diluted, and plated on duplicate TGY plates. All plates were wrapped in parafilm and were incubated at room temperature (22–25 °C) with one set under 57 Gy/h (^137^Cs; GammaCell 40, J. L. Shepard and Associates) and the other outside of the irradiator. After 5.7 days (2668.65 min) of chronic radiation exposure, images were taken of resulting plates.

### RNA extraction and Northern blotting

Total RNA extraction and Northern blotting analysis were performed as previously described^37,38^. The cells were collected and suspended with 1 mL TRIzol reagent (Invitrogen) and lysed by bead-beating (Bio Spec Products Inc.) with two 100 s pulses. Total RNA was then extracted with 300 µL chloroform-isoamyl alcohol (24:1), precipitated with 300 µL isopropanol and 1 µL GlycoBlue (Thermo Fisher Scientific) overnight at − 20 °C, and the resulting pellet dissolved with 30 µL nuclease-free water. For Northern Blotting, RNA samples were loaded into a 10% polyacrylamide gel for total RNA electrophoresis under denaturing conditions and then were transferred onto a positively charged membrane (Hybond N+; GE Life Sciences) in TBE buffer for 18 h. The probes were radiolabeled with γ-^32^P for hybridization. All the oligonucleotides that were used in this study are listed in Table S9. Membranes were hybridized with radio-labeled probe for 16 h at 42 °C before wash and imaging using a Typhoon FLA 700 (GE Health Life Science). Band intensities were quantified using ImageJ analysis^71^ or CLIQS software (Total Lab). Full images for the Northern blots are presented in Fig. S10.

### RNA half-life determination

RNA half-life determination was performed as described previously^72,73^. Briefly, triplicate cultures of *D. radiodurans* R1 (WT) and PprSKD were grown to exponential phase (OD_600_ = 0.8) and incubated with 250 µg/mL of rifampicin (VWR) in DMSO. After a specific amount of time (0–60 min), 5 mL of culture was removed and snap frozen in liquid nitrogen. RNA was then extracted from samples as described above, and *pprM* transcript levels determined by Northern Blotting analysis. Half-life was determined from the normalized intensities of *pprM* from t_1/2_ = ln(2)/k with k as the negative slope of the ln[mRNA] over time^72^.

### MS2 affinity purification coupled with RNA sequencing (MAPS)

Full methods can be found in the “Supplemental Information”. In brief, determination of PprS’s possible mRNA targets was performed according to a protocol published previously^57^. The MS2 protein binding site sequence (MS2BD) was added to the 5′ end of the *PprS* sequence and cloned into the pRADgro plasmid (Tables S8 and S9) for expression in wild-type *D. radiodurans* R1^58^. A negative control was also made that did not contain the PprS sequence, only the MS2BD (pRADgro-MS2). *D. radiodurans* expressing the pRADgro-MS2-PprS plasmid were cultured to late-exponential phase (OD_600_ = 1) and collected for total RNA extraction. Total RNA was extracted as mentioned above. To extract the mRNAs associating with PprS from the total RNA sample, 2 µg of MS2 coat protein fused with maltose binding protein (MS2-MBP)^57^ expressed from *E. coli* was incubated with 100 µL of total RNAs (~ 1 µg/µL), containing MS2BD-PprS transcripts, for 1 h at 4 °C. The *MS2BD-PprS*-MBP protein complexes were washed and eluted before precipitation with equal volume of isopropanol and 10 µL GlycoBlue (Thermo Fisher Scientific) overnight at − 20 °C. Following precipitation, RNA was washed with cold 75% ethanol, resuspended in 20 µl nuclease-free water, and prepared for sequencing (described below).

### Transcriptome analysis

The cDNA libraries were prepared from total RNAs using NEBNext RNA First Strand Synthesis Module (NEB E7525L) and NEBNext DNA Library Prep Master Mix Set for Illumina (NEB E6040L). Total RNAs were prepared as mentioned above from irradiated samples and RNAs from MS2-MBP co-immunoprecipitation. cDNA libraries were then analyzed with Illumina NextSeq 500 single-end platform. A total of around 6,000,000 reads were mapped to the genome for each sample, and reads that mapped to rRNA or tRNA were then excluded from our analysis. All sequenced reads were trimmed to remove the adapter sequence for mapping to the *D. radiodurans* R1 genome (GenBank accession numbers NC_001263.1 and NC_001264.1) with Bowtie2 Aligner. Differential expression of genes was normalized and calculated by DEseq2 algorithm^74^. Genes with expression level changes more than twofold increased or reduced with adjusted *p*-value < 0.05 were considered as significantly differentially expressed.

### Electrophoretic mobility shift assay (EMSA)

PprS RNA with or without designed mutations was synthesized (from Integrated DNA Technologies, Inc.) DNA templates of *pprM* 5′UTR (− 33 bp to + 100 bp from the start codon) with or without designed point mutation was also synthesized for in vitro transcription using MEGAscript T7 Transcription Kit (AM1334, ThermoFisher) (sequences in Table S9). The RNA samples were then cleaned up using RNA Clean & Concentrator Kits (R1013, Zymo Research) and the RNA concentrations were determined using Nanodrop. Purified *pprM* 5′UTR RNA samples were then radiolabeled with γ-^32^P by T4 polynucleotide kinase (New England BioLabs). 1 pmol of radiolabeled *pprM* mRNA fragment and 0–100 pmol of unlabeled PprS sRNA were incubated in a 12 μL reaction buffer [20 mM Tris–HCl (pH 8.0), 1 mM MgCl_2_, 20 mM KCl, 10 mM Na_2_HPO_4_–NaH_2_PO_4_ (pH 8.0)), 10% glycerol]. The samples were denatured at 75 °C for 10 min and further incubated in a water bath at 37 °C for 1.5 h. The reactions were separated in an 8% nondenaturing polyacrylamide gel (0.5 × TBE, 8% wt/vol acrylamide-bisacrylamide, 5% glycerol, 0.25% ammonium persulfate, and 0.001% TEMED) with 0.5 × TBE running buffer at 4 °C for 4–6 h. The gels were then placed on a blotting paper (WHA3030861, Whatman) and dried at 80 °C for 60 min. Radioactive bands were visualized using a Typhoon FLA 700 (GE Health Life Science) and analyzed. The fraction bound was then calculated based on the ratio of the intensity of the RNA-RNA complex to the total intensity under each concentration as measured by ImageJ analysis^71^ or CLIQS software (Total Lab). The equilibrium dissociation constant (K_d_) was determined as described before^75^.

### Quantification RT-PCR analysis (RT-qPCR)

Following RNA extraction, DNA contamination was removed using DNase1 (NEB) and heat deactivated at 75 °C. DNase was removed using RNA/DNA Clean and Concentrate Kit 5 (Zymo Research). RNA was denatured at 65 °C for 5 min before annealing with random hexamer primers at room temperature for 10 min. RNA was converted to cDNA using SuperScript III (Thermo Fisher Scientific) at 50 °C for 60 min, followed by heat inactivation at 70 °C for 15 min. Primers for qPCR primers were designed using PrimerQuest online tool (IDT) or previously published^38,45^ and can be found in Table S9. Primer efficiencies were established across 4 dilutions of cDNA using the Thermo Fisher qPCR efficiency calculator. Primer efficiencies ranged between 95 and 109%. All qPCR reactions prepared using the SyberGreen POWER qPCR mix (Life Technologies) in 10 µL reactions according to manufactures protocol in MicroAmp Optica 398-well Reaction Plates (Life Technologies). Reactions were performed in biological triplicate on a ViiA 7 light thermal cycler (Agilent) for three separate experiments. All samples were denatured at 95 °C for 10 min and, annealed at 58 °C and extended at 68 °C for 40 cycles. Transcript fold changes were calculated using the DeltaDelta CT method^76^.

## Supplementary Information

Supplementary Dataset S1

Supplementary Information

## Data Availability

The data discussed in this publication are publicly available. The mass spectrometry proteomics data have been deposited to the ProteomeXchange Consortium via the PRIDE^77^ partner repository with the dataset identifier PXD026633. The RNA-sequencing data have been deposited to NCBI's Gene Expression Omnibus (GEO)^78,79^ and are accessible through GEO Series accession number GSE176207
