# Supplementary material for: A small RNA regulates pprM, a modulator of pleiotropic proteins promoting DNA repair, in Deinococcus radiodurans under ionizing radiation
Source: Sci Rep. 2021 Jun 21;11:12949. doi: 10.1038/s41598-021-91335-8 (PMC8217566; doi:10.1038/s41598-021-91335-8)
Supplement: Supplementary file 2 — Supplementary Information [file 41598_2021_91335_MOESM2_ESM.pdf]

Supplementary Information for

A small RNA regulates pprM, a modulator of pleiotropic proteins promoting DNA repair, in *Deinococcus radiodurans* under ionizing radiation

Jordan K. Villa, Runhua Han, Chen-Hsun Tsai, Angela Chen, Philip Sweet, Gabriela Franco, Respina Vaezian, Rok Tkavc, Michael J. Daly, Lydia M. Contreras*

Corresponding Author: Lydia M. Contreras
Email: lcontrer@che.utexas.edu

This PDF file includes:

- Supplementary text
- Figures S1 to S10
- Legends for Dataset S1 (Tables S1 to S9)
- SI References

Other supplementary materials for this manuscript include the following:

- Dataset S1 containing Tables S1 to S9

Supplementary Information Text

Supplemental Materials and Methods

MS2 affinity purification coupled with RNA sequencing (MAPS)

Determination of the possible mRNA targets of PprS was performed according to a protocol published previously[1]. In brief, the MS2 protein binding domain (MS2BD) sequence was added to the 5' end of the *pprS* sequence and cloned into the pRADgro plasmid (pRADgro-PprS-MS2) (Table S8 and S9) for expression in *D. radiodurans* [2]. A negative control was also made that did not contain the PprS sequence, only the MS2BD (pRADgro-MS2). *D. radiodurans* expressing the pRADgro-MS2-PprS plasmid were cultured to exponential phase (OD_{600} = 1) and collected for total RNA extraction. Total RNA was extracted as mentioned above.

To use for affinity purification, MS2 coat protein fused with maltose binding protein (MS2-MBP)[1] was expressed in *E. coli* in a 100 mL culture induced with 1 mM IPTG at OD_{600} = 0.5 for four hours. Cells were then collected and resuspended in 10 mL column buffer (20 mM Tris-HCl, 200 mM NaCl, 1 mM EDTA, 10 mM β–mercaptoethanol pH 7.4) and lysed using a probe sonicator (XL-2000 Microson ultrasonic liquid processor; QSonica) on ice. After sonication, the lysate was treated with DNAse for 1 hour at 4°C and supernatants was collected after centrifugation. To purify MS2-MBP from lysates, 100 µL of amylose magnetic beads (New England Biolabs) added to 500 µL of lysates and incubated for 2-3 hours at 4°C. Sample was then was washed with 1 mL wash buffer (column buffer + 0.1 mM maltose) three times. After application of a magnet (Thermofisher scientific, Magjack rack), MS2-MBP protein was eluted with 50 µL elution buffer (column buffer + 10 mM maltose). Purified MS2-MBP was confirmed by SDS-PAGE and the concentration was determined by Bradford assay.

To extract the mRNAs associating with PprS from the total RNA sample, 2 µg of MBP protein was incubated with 100 µL of total RNAs (~1 µg/µL), containing MS2BD-PprS transcripts, for 1 hour at 4°C. This mixture was then incubated with amylose magnetic beads (New England Biolab) for another 2 hours and supernatants were removed after magnets were applied. The beads were washed three times with wash buffer and the *MS2BD-PprS* MBP protein complexes were eluted with 50 µL elution buffer. Total RNA was precipitated with equal volume of isopropanol and 10 µL GlycoBlue™ overnight at -20°C, washed with 1 mL. cold 75% ethanol, and resuspended in 20 uL nuclease-free water. RNA samples were then prepared for sequencing (described in the main text).

5' RACE

Rapid amplification of cDNA ends (RACE) to determine the 5' end of *pprM* transcript was performed as previously published[3] using the FirstChoice RLM-RACE kit (Ambion) according to the manufacturer's protocol. Briefly, total RNA was extracted from *D. radiodurans* R1 as described above. 10 µg of RNA was ligated to the kit-provided 5'RACE adapter using T4 RNA ligase at 37˚C

for 1 hour before reverse transcription using Moloney murine leukemia virus (MMLV) reverse transcriptase and random decamer primers (N12) at 42°C for 1 hour. The resulting cDNA was then PCR amplified (primers in Table S9) and sequenced to determine the transcription start site.

Proteomic Analysis

D. radiodurans R1 and PprSKD cultures were grown to an OD_{600} of ~1.2 (50 mL cultures) in biological triplicates and incubated at 4˚C for two hours before collecting the cell pellet and storing at -80˚C, to mimic the conditions of irradiated *D. radiodurans* samples (described above). For the time-course experiment, biological triplicate *D. radiodurans* R1 cultures were grown to exponential phase and irradiated with 10 kGy acute IR (described in main text). Following irradiation, cultures were recovered for 0-4 h at 32˚C before protein extraction.

For each experiment, protein lysis was obtained using a previous method[3] of sonication at 10 V for 1 min for three bursts with 5 min rest on ice in between each pulse. The soluble protein was concentrated via an acetone precipitation for 16 hrs at -20˚C, followed by resuspension in 200 µL of SDS-PAGE sample buffer. This resuspension was directly loaded onto a 12% SDS-PAGE gel and run 2 mm into the stacking layer. The resulting coomassie stained gel band was cut and in-gel trypsin digested using 20 ng/µL trypsin (Pierce™ Thermo Fisher Scientific; catalog #90057) following previously published protocols[3,4]. Digested samples in 0.1% formic acid were injected into a Thermo Orbitrap Fusion hybrid linear ion trap FT-MS with Dionex 3000 nanospray UPLC and run for 2 hrs per sample at the ICMB Proteomics Facility. Resulting peptide fragments were searched against the Uniprot *D. radiodurans* R1/ATCC 13939 database using Sequest HT in Proteome Discoverer 1.4. Resulting protein spectral counts were analyzed using the Scaffold4 program with greater than 99.0% probability and a minimum of two peptides at 95% peptide probability. Normalized spectral abundance factor (NSAF) was calculated for each protein spectral count using Scaffold4 program and differential expression of proteins was calculated using the R DEP Package[5].

Supplementary Figures

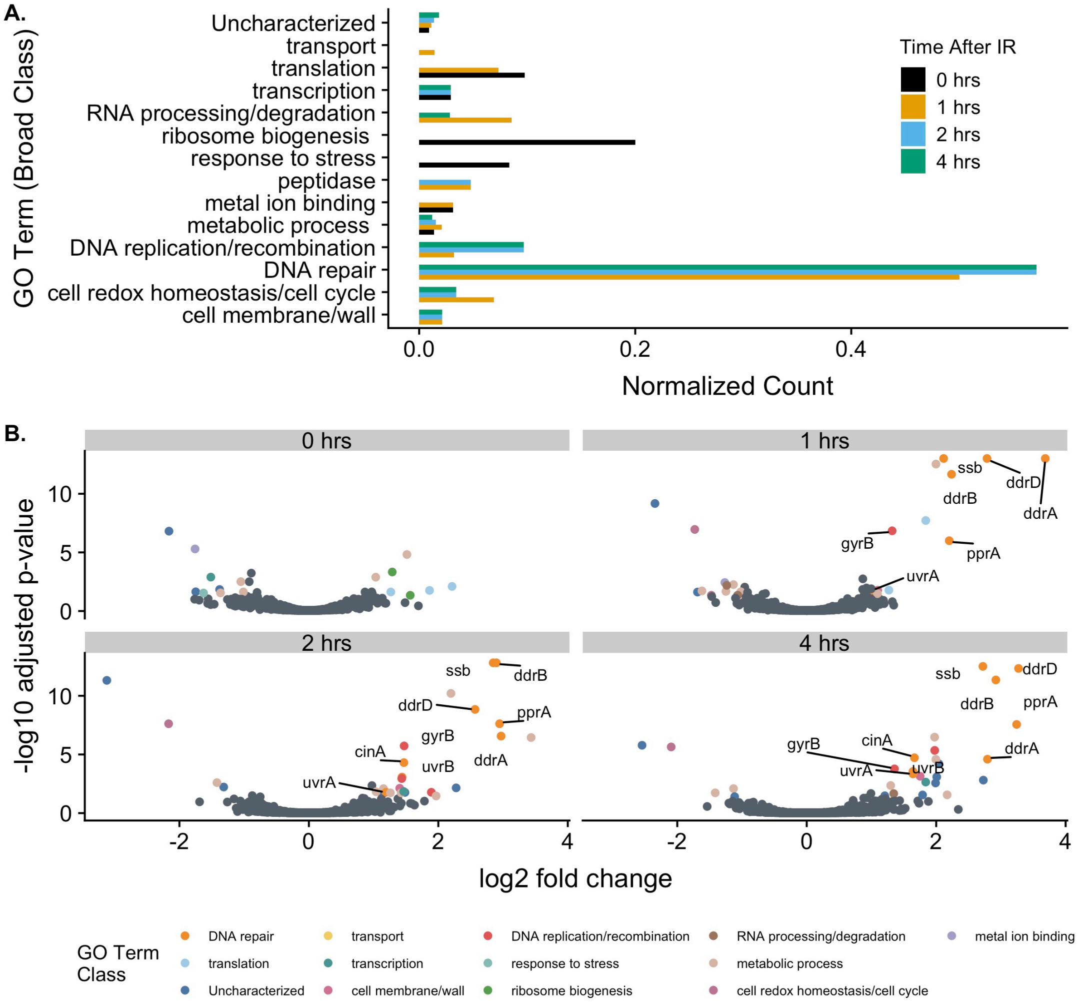

Figure S1. Proteomics time-course shows rapid upregulation of RDR pathway. (A) Normalized count of proteins of the significantly differentially expressed (adjusted *p*-value <= 0.05 and log2 fold-change > 1 or < -1) from each GO term pathway class after 10 kGy of acute IR at varying times of recovery. Normalized count was calculated by normalizing the number of significantly differentially expressed proteins in each GO term pathway to the total number of proteins within that term. GO Enrichment analysis using PANTHER tools demonstrated significant enrichment of DNA repair and response to radiation for comparisons of 1-4 hrs after IR. (B) Volcano plot of the significantly differentially expressed proteins at varying times of recovery from 10 kGy acute IR. Proteins in the RDR pathway are labeled with text and significantly differentially expressed proteins are colored with the different pathways from GO terms. Fold-changes and *p*-values were determined from biological triplicate samples.

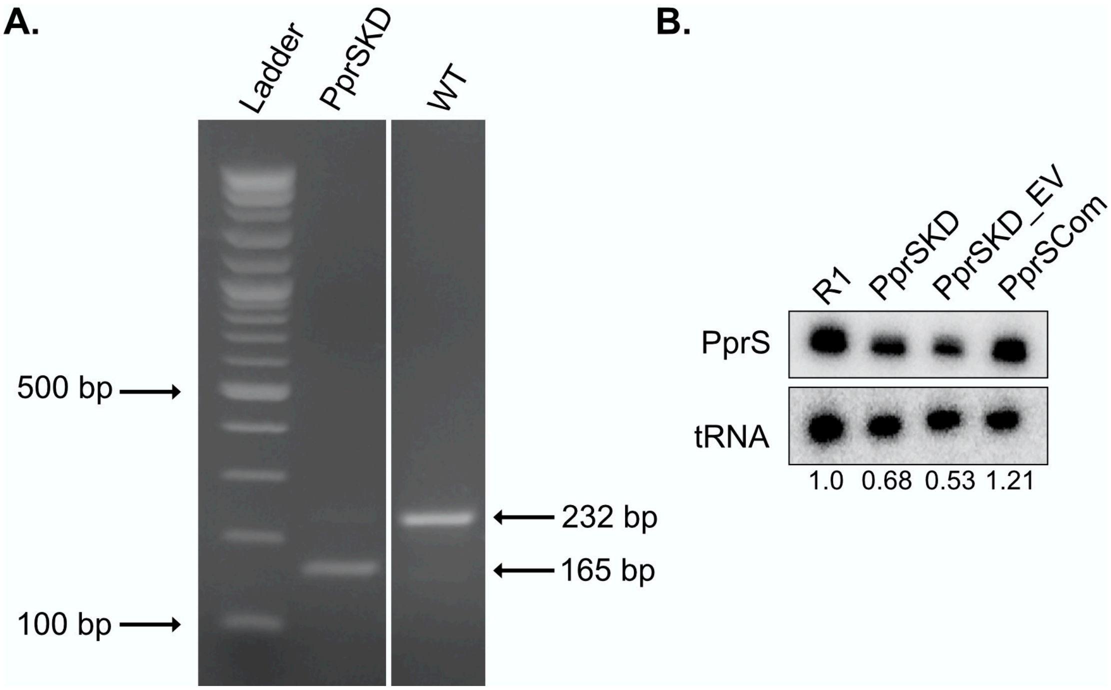

Figure S2. Confirmation of knockdown of PprS in *D. radiodurans* by (A) genomic PCR where the 165 bp band demonstrates the successful mutant construction. The gel has been cropped to remove extra lanes. (B) Northern blotting analysis demonstrates a ~2-fold reduction in PprS expression in the KD strain. PprS levels are restored in the complement strain (PprSKD + pRADgro-PprS) in comparison to PprSKD with the empty vector (pRADgro) (PprSKD_EV). Values under the blot represent the normalized PprS levels (normalized to tRNA). Northern blot has been cropped to show specific bands of interest from the same membrane.

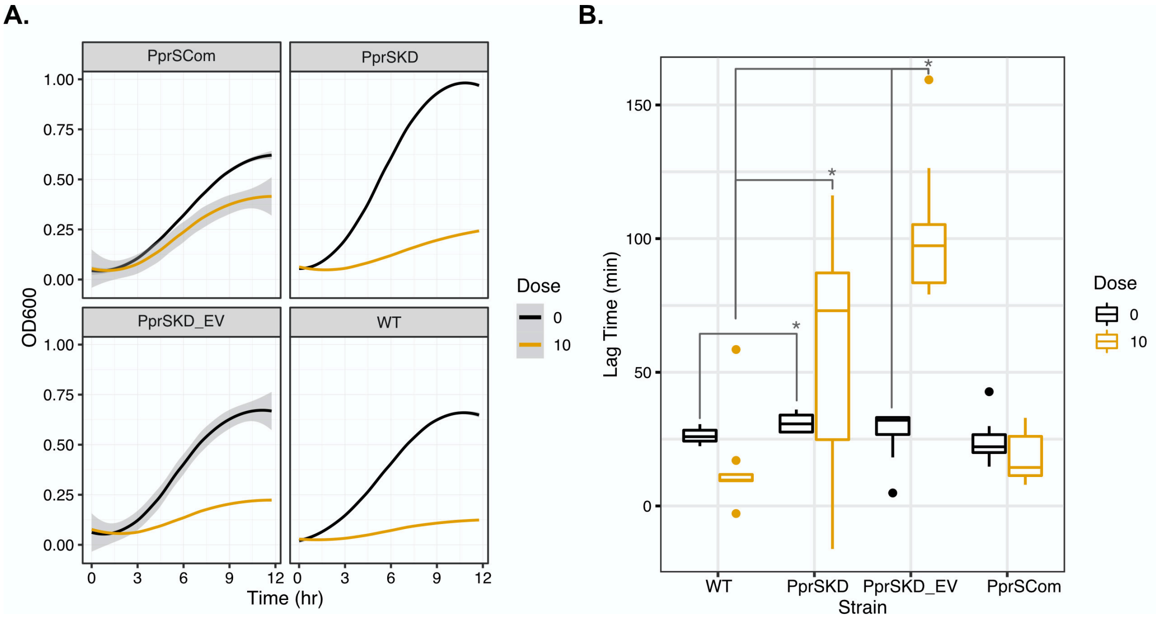

Figure S3. PprSKD demonstrates increase in lag time following 10 kGy IR compared to WT. (A) Growth Curves of *D. radiodurans* strains after exposure to 0 or 10 kGy acute IR. Lines represent average absorbance at OD_{600} and shading represents 95% confidence interval from triplicate biological replicates. (B) Boxplot lag times calculated from the growth curves in (A). Differences in lag time were determined to be significant using a Welch Two Sample t-test (* = p-value < 0.05).

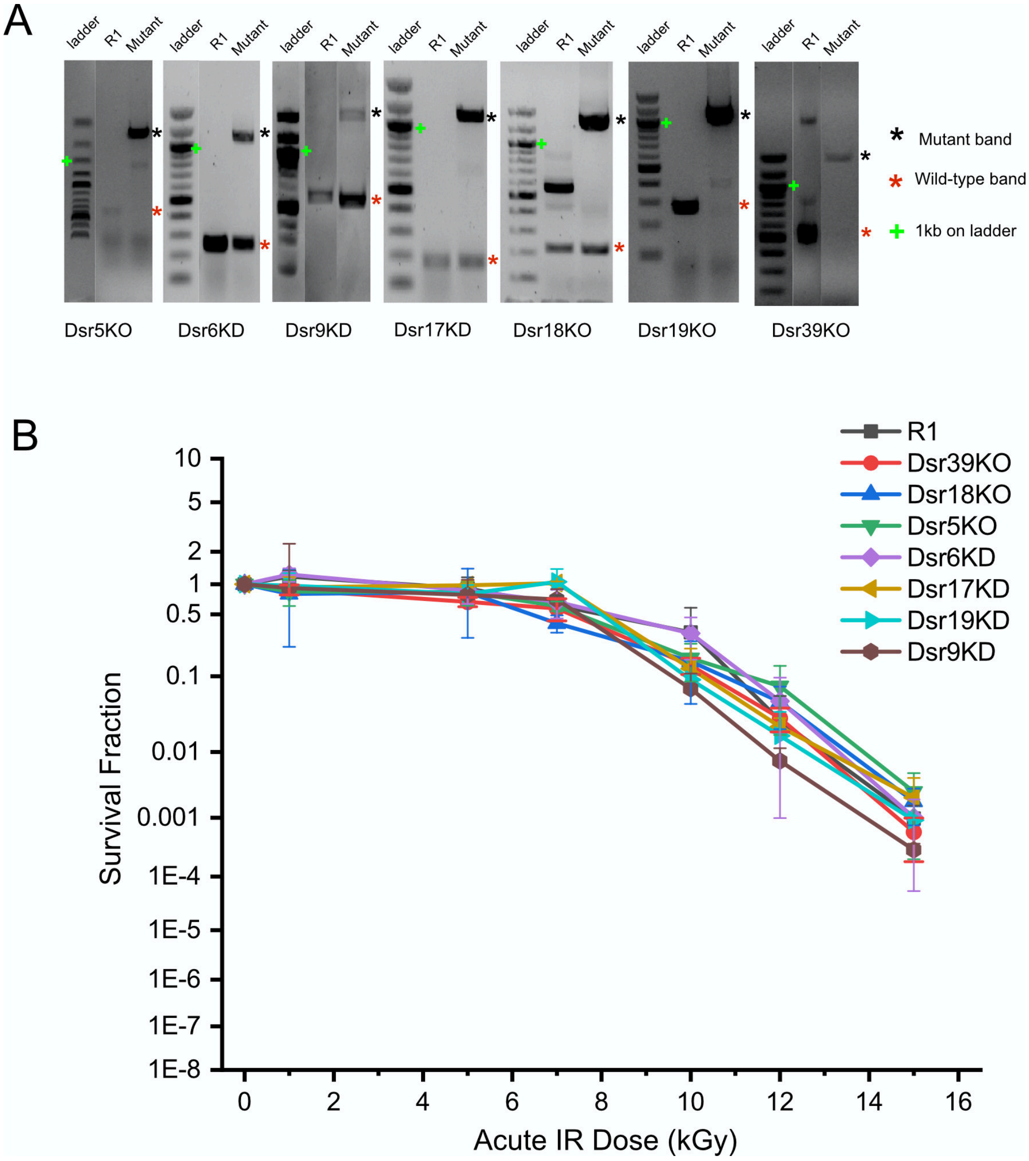

Figure S4. Knockout (KO) or knockdown (KD) of other sRNAs do not demonstrate decrease in survival to acute IR. (A) Genomic PCR confirmations of sRNA knockout (KO) or knockdown (KD). (B) Survival curve of sRNA knockouts (KO) and knockdowns (KD) under acute IR. Error bars are standard deviations of biological triplicate samples.

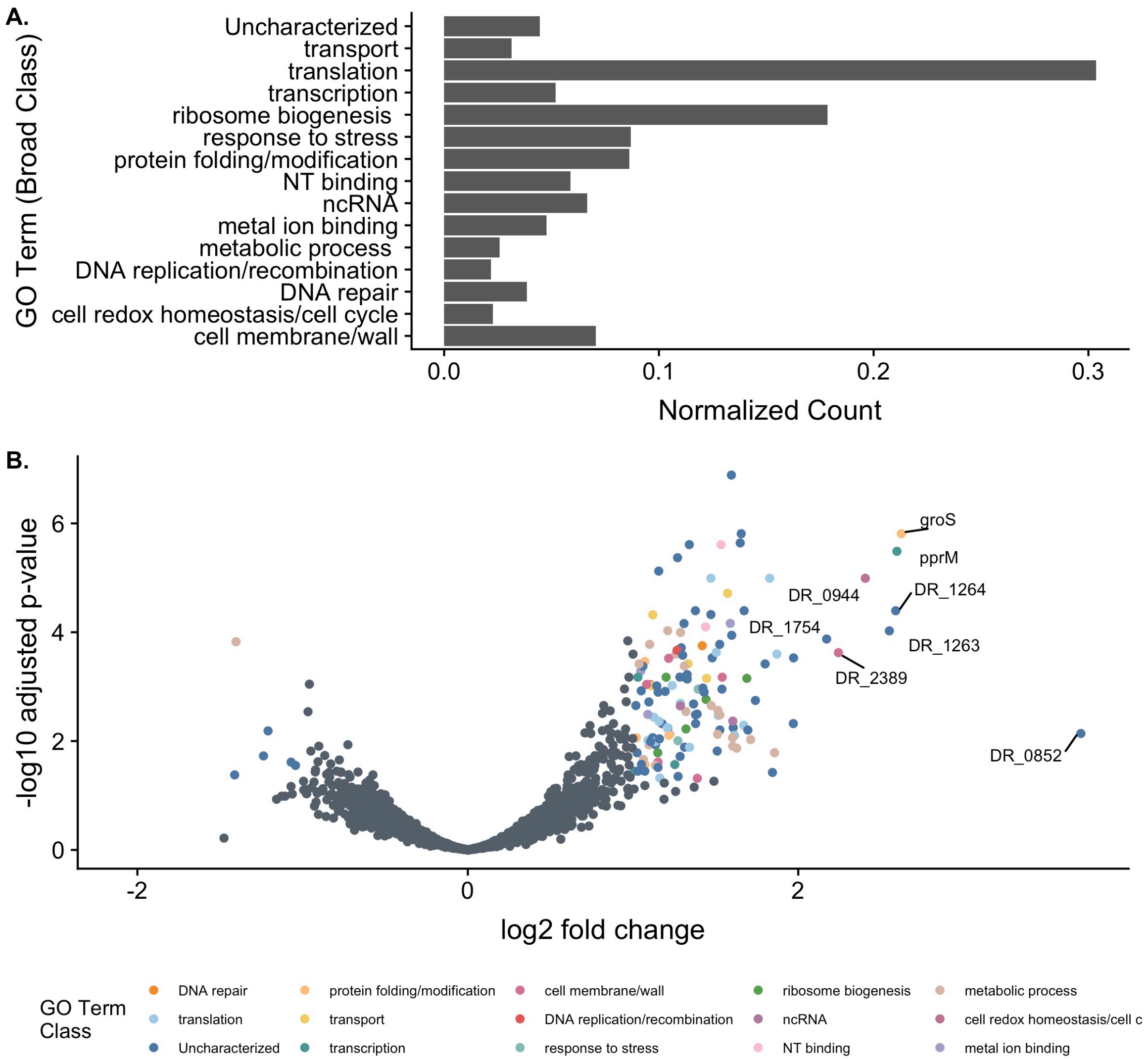

Figure S5. MS2-Affinity Purification coupled with RNA-Sequencing (MAPS) Analysis Predicts Putative PprS Targets related to DNA repair and translation/transcription. (A) Normalized count of the broad pathway terms of the significantly differentially enriched (adjusted *p*-value <= 0.05 and log2 fold-change > 1 or < -1) transcripts from MAPS analysis. Normalized count was calculated by normalizing the number of significantly differentially expressed proteins in each GO term pathway class to the total number of proteins within that term. GO Enrichment Analysis using PANTHER tools demonstrates significant enrichment of translation and gene expression GO terms. (B) Volcano plot of the significantly differentially enriched transcripts from MAPS analysis. Transcripts that were significantly enriched with log2 fold change > 2 are labeled with text and significantly differentially expressed proteins are colored with the different pathways from the GO term classes.

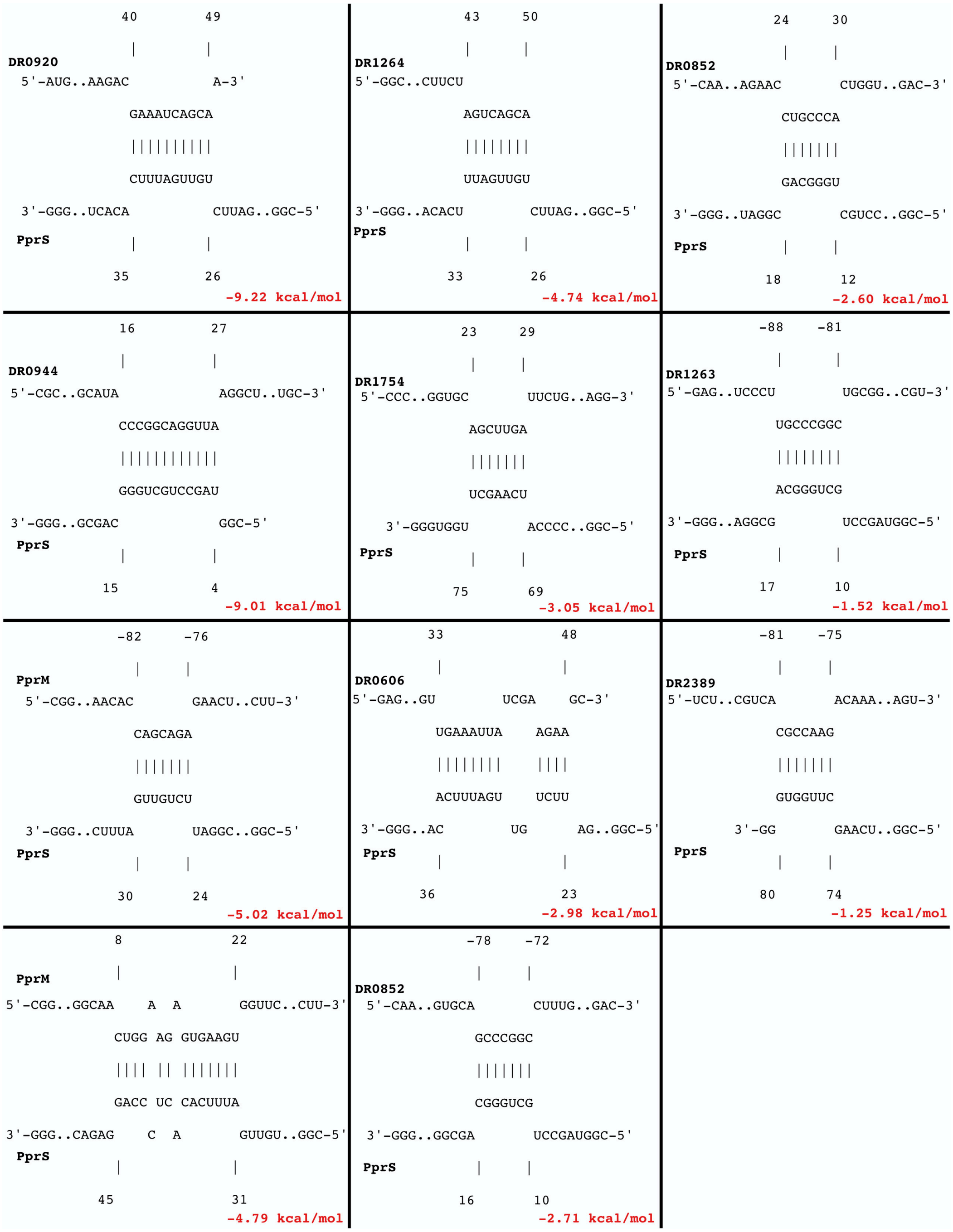

Figure S6. IntaRNA Predictions for the top 10 MAPS enriched mRNA targets. Regions of the predicted mRNA targets, composed of 150 nt upstream and 50 nt downstream the annotated start codon were run using default IntaRNA settings, but permitting top 2 interactions for each pair. Two RNAs regions contained two predicted binding regions (pprM and DR_0852) and one RNA region (DR_1261) did not produce any predicted binding regions. In each figure, the position of binding interaction for the mRNA targets is indicated relative to the location of the start codon; for PprS these positions are relative to the transcription start site. Numbers in red are the predicted binding energies provided by IntaRNA. Note that the corrected start codon position was utilized for *pprM* which places one of the predicted binding regions (location -76 to -82 from the start codon) outside of the transcript (as determined by 5' RACE). Thus, this prediction was not used in the rest of our analysis.

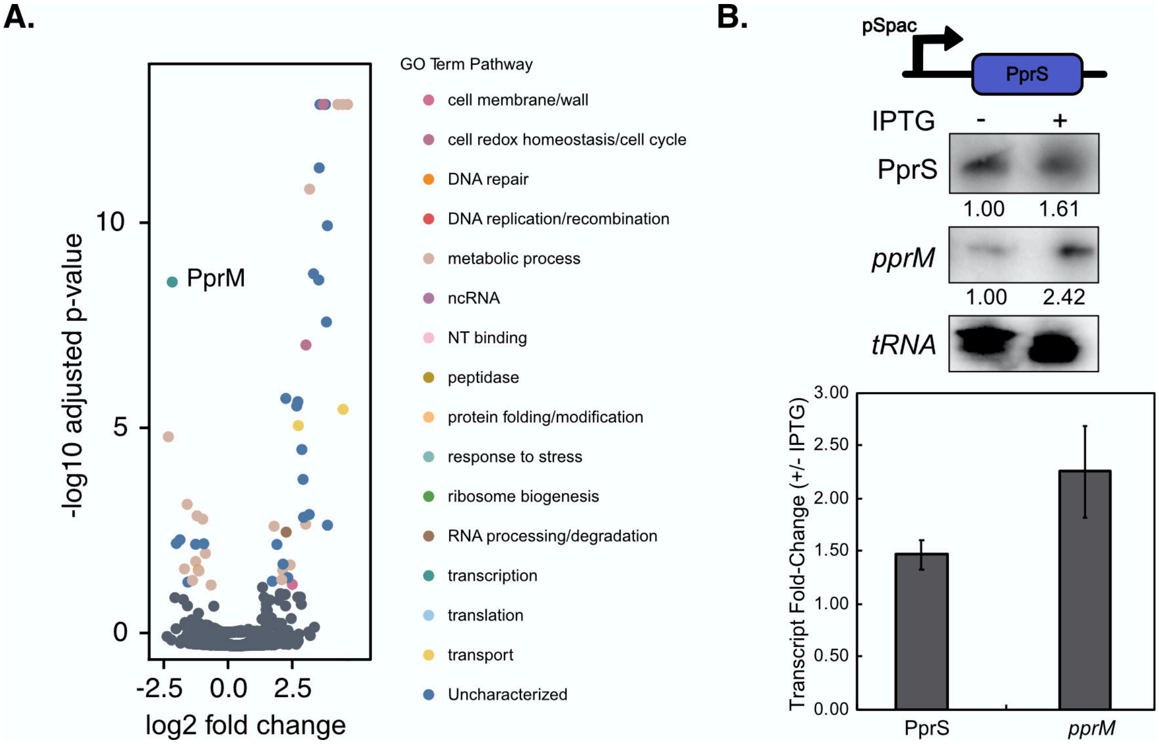

Figure S7. Knockdown of PprS reduces PprM protein levels while inducible expression of PprS stabilizes native *pprM* transcript. (A) Volcano plot of significant differentially (padj < 0.05 and log2 fold-change > 1 or < -1) expressed proteins colored by GO terms. PprM (labeled by text) was at significantly lower levels in the PprSKD strain than WT. (B) Representative Northern blotting analysis of PprS and *pprM* in *D. radiodurans* PprSKD expressing PprS under an inducible pSpac promoter (induced with 1 mM IPTG for 8-16 hrs). Numbers below the gel image represent the normalized fold change (PprS or *pprM* expression normalized to tRNA, then normalized to the uninduced data). Northern blots have been cropped to show specific bands of interest, but are from the same membrane for each figure. Average fold-changes (induced/uninduced) for each transcript are shown in bar plot below. Error bars represent standard deviation from triplicate independent experiments.

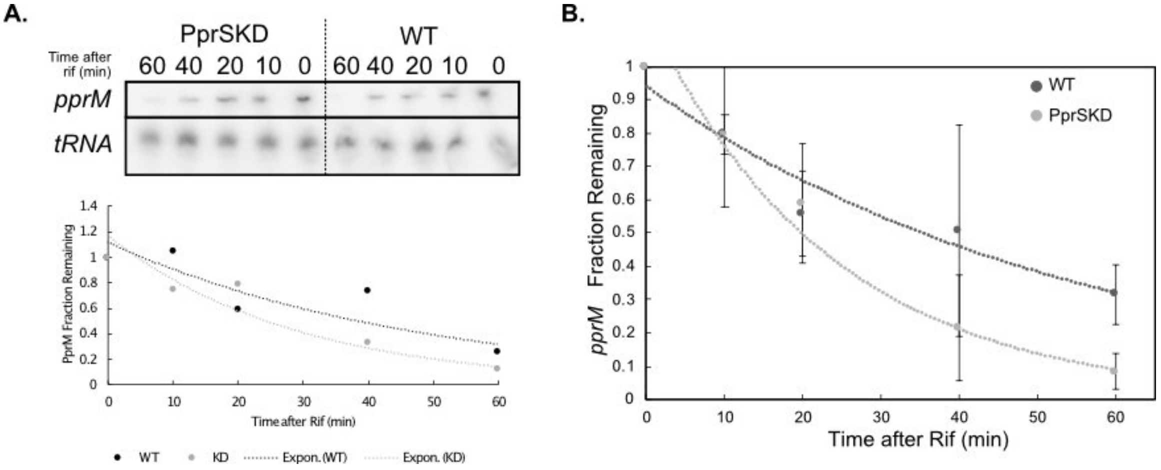

Figure S8. Representative Northern blot for *pprM* half-life determination. *D. radiodurans* samples were treated with 250 μg/mL rifampicin and RNA samples collected at varying incubation times. Intensities are normalized to tRNA and t = 0 min (before rifampicin addition) and half-life determined from $t_{1/2}$=ln(2)/k with k as the negative slope of the ln[mRNA] over time. (A) Representative Northern blot to determine mRNA half-life. Northern blot has been cropped to show specific bands of interest, but are from the same membrane. Northern blotting analysis was performed in biological triplicate with one representative shown here. (B) Plot demonstrative the average *pprM* fraction remaining for the three biological replicate samples. Half-life was determined for each replica independently and the three half-lifes were then averaged to determine the average half-life of *pprM* for the two *D. radiodurans* strains.

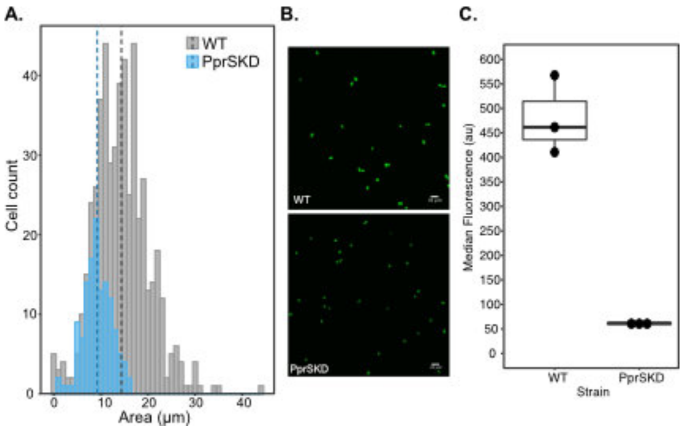

Figure S9. *D. radiodurans* PprSKD has decreased cell size (A-B) and decreased fluorescence (B-C) compared to WT. (A) Histogram of the area of cells (of at least two biological replicates and four imaged areas) from (B) fluorescent microscopy of cells expressing a constitutive GFP reporter. Dotted lines show mean area of cells (14 μm versus 9 μm between WT and PprSKD, respectively; *p*-value from two-tailed Students t-test = 2.2×10^{-16}). (C) Median fluorescence of cells expressing the same constitutive reporter measured via flow cytometry. Dots represent biological replicates; two-tailed Student's t-test demonstrates a significant (p-value < 0.05) difference in average median fluorescence between strains.

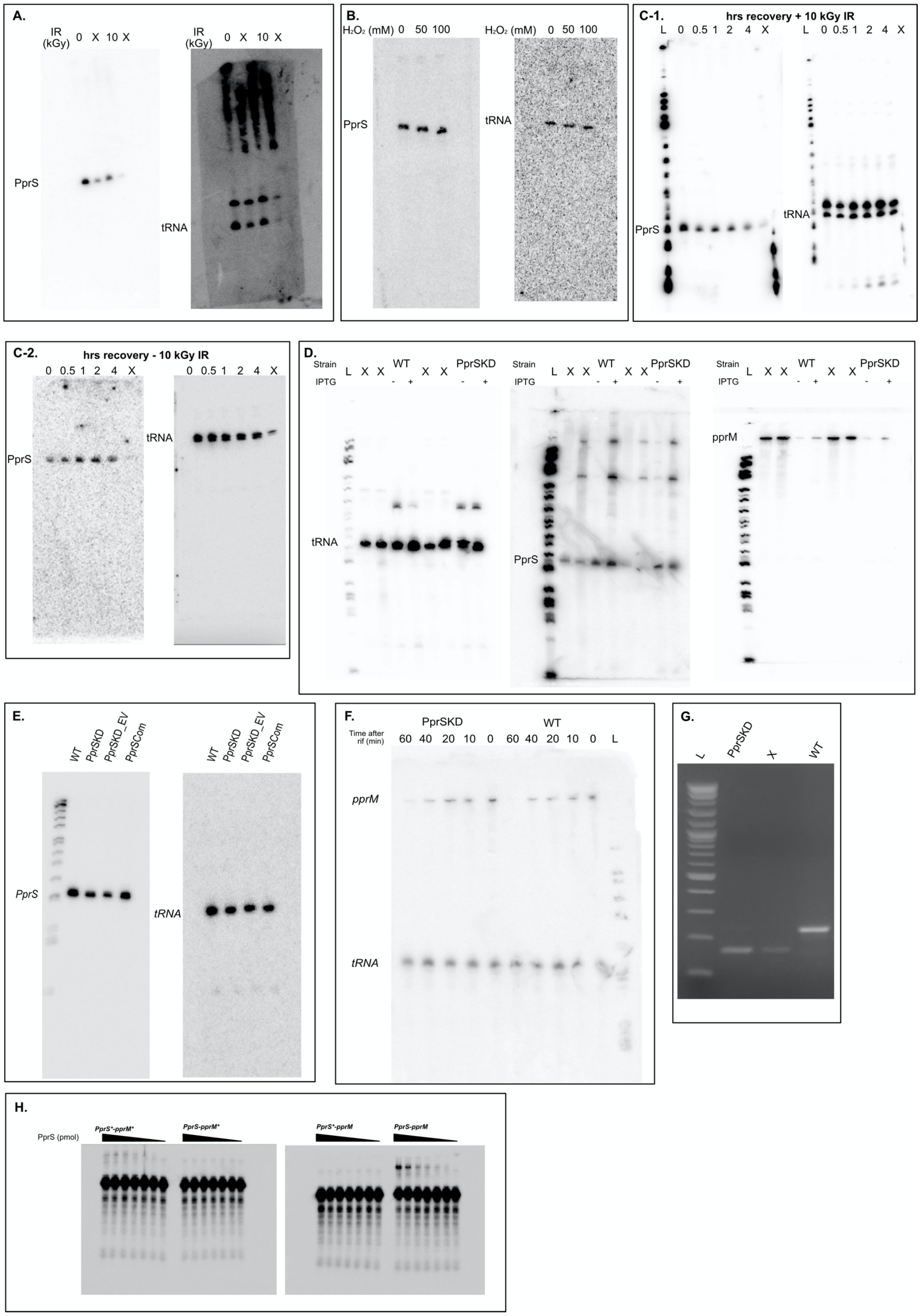

Figure S10. Full images for Northern blots, PCRs, and EMSAs presented in figures. In each Northern blot, irrelevant lanes are labeled with an X. The bands that were cropped for the manuscript are labeled. For some blots both the PprS/*pprM* probe was able to be visualized in same exposed image as the tRNA loading control. In others, the blot was stripped before probing for the next transcript. Lanes with PhiX174 (HinfI digest) ladder is marked with L. Images from the same membrane are boxed together. Full Northern blots are presented for (A) Figure 1A, (B) Figure 1B, (C-1 and C-2) Figure 1C, (D) Figure S7B, (E) Figure S2B, and (F) Figure S8A. (G) Figure S2A full genomic PCR gel for PprSKD confirmation where L is the Neb 2 log ladder and X is the removed lane. (H) The full gels for EMSA of *pprM* and PprS binding from Figure 3C.

Dataset S1 (separate file).

Excel file containing the following tables:

Table S1. Significantly differentially expressed proteins from time-course proteomics data from *D. radiodurans* R1 (WT) during recovery from 10 kGy acute IR.

Table S2. GO Term Enrichment analysis results for all transcriptomics and proteomics data performed by PANTHER Tools GO Enrichment analysis.

Table S3. MS2-affinity purification coupled with RNA-Sequencing (MAPs) data of significantly enriched PprS targets.

Table S4. Significantly differentially expressed proteins from proteomics data of *D. radiodurans* PprSKD versus WT at sham (not irradiated) conditions

Table S5. Significantly differentially expressed transcripts from transcriptomics data of *D. radiodurans* PprSKD versus WT at sham (not irradiated) conditions.

Table S6. Significantly differentially expressed transcripts from transcriptomics data of *D. radiodurans* WT during recovery from 10 kGy acute IR.

Table S7. Significantly differentially expressed transcripts from transcriptomics data of *D. radiodurans* PprSKD during recovery from10 kGy acute IR.

Table S8. List of strains and plasmids used in this study.

Table S9. List of primers and sequences used in this study.

SI References

1. Lalaouna, D. & Massé, E. Identification of sRNA interacting with a transcript of interest using MS2-affinity purification coupled with RNA sequencing (MAPS) technology. *Genomics Data* **5**, 136–138 (2015).
2. Misra, H. S. *et al.* An exonuclease I-sensitive DNA repair pathway in Deinococcus radiodurans: A major determinant of radiation resistance. *Mol. Microbiol.* **59**, 1308–1316 (2006).
3. Villa, J. K. *et al.* A genome-wide search for ionizing-radiation responsive elements in Deinococcus radiodurans reveals a regulatory role for the DNA gyrase subunit A gene's 5' untranslated region in the Radiation and Desiccation Response. *Appl. Environ. Microbiol.* **83**, (2017).
4. Shevchenko, A., Tomas, H., Havliš, J., Olsen, J. V. & Mann, M. In-gel digestion for mass spectrometric characterization of proteins and proteomes. *Nat. Protoc.* **1**, 2856–2860 (2007).
5. Zhang, X. *et al.* Proteome-wide identification of ubiquitin interactions using UbIA-MS. *Nat. Protoc.* **13**, 530–550 (2018).
